# Mental Health and the Role of Physical Activity During the COVID-19 Pandemic

**DOI:** 10.3389/fpsyg.2021.759987

**Published:** 2021-10-20

**Authors:** Xianfeng Ai, Jingjing Yang, Zhibin Lin, Xiaohong Wan

**Affiliations:** ^1^School of International Education, Wuhan Sports University, Wuhan, China; ^2^Macao Institute for Tourism Studies, Macao SAR, China; ^3^Durham University Business School, Durham University, Durham, United Kingdom; ^4^School of Journalism and Communication, Wuhan Sports University, Wuhan, China

**Keywords:** mental health, COVID-19 pandemic, coping behavior, integrated framework, physical activity

## Abstract

The COVID-19 pandemic and its related public health restrictions are having an increasingly serious impact on mental health, and measures need to be taken to curb this trend. The positive relationship between physical exercise and mental health has been well-established, but during the COVID-19 pandemic, with various restrictions, the space and facilities for physical exercise are limited. This article explores the relationship between physical exercise and mental health during the COVID-19 pandemic based on the latest research findings published in 2019–2021. We offer a novel model that consists of three central arguments. First, physical exercises during COVID-19, especially supervised exercises, are conducive to enhancing happiness and improving mental health. Second, physical exercise reduces people's anxiety, sadness and depression during the COVID-19 pandemic. Third, the maintenance and improvement of mental health are related to the intensity and frequency of physical exercise. Intensive and frequent physical exercise are conducive to maintaining mental health. Finally, this article proposes important directions for future research.

## Introduction

Physical activities, personal contact with colleagues, friends and family have been limited due to public health measures to curb the spread of COVID-19, such as quarantine, lockdown, self-isolation, and maintaining social distance. People experienced a general decline in well-being, deterioration in mental health, and an increase in psychological distress such as stress, anxiety, depression, and feelings of isolation (Chtourou et al., 2020; Gupta et al., 2021). This negative impact may, in turn, affect compliance with public health measures (Puyat et al., 2020). Hence, there is a need to explore and identify alternative and complementary activities that promote mental health and well-being during COVID-19.

It is well-established that physical activity or exercise is essential to improve and/or maintain physical and mental health and improve the quality of life (Warburton et al., 2006; Bird et al., 2021; Vancini et al., 2021). Various physiological and psychological mechanisms have been proposed to explain the positive effects of physical activity (Anderson and Shivakumar, 2013; Stubbs et al., 2017). Physiologically exercise may increase the levels of hormones, endorphins and Brain-derived Neurotrophic Factor (BDNF), which could make people feel happy and less stressed (Harber and Sutton, 1984). Moreover, psychologically, exercise gives people the opportunity to have “time out” from the stressor, and regular exercise improves self-efficacy to overcome the difficulties they face (Petruzzello et al., 1991).

The COVID-19 pandemic has heightened people's attention to the positive effects of exercise on physical health. During the pandemic, the news media disseminates a great deal about the therapeutic effects of physical activities on physical health and people with mild coronavirus infection. Regular and long-term physical exercise enhances the body's immune surveillance capabilities, acts as an anti-inflammatory agent, reduces the risk of developing numerous chronic diseases, and improves the general level of physical health and disease prevention and resistance (Shephard et al., 1991).

Initial evidence has shown that individuals can maintain and promote mental health through exercise during the pandemic (Gupta et al., 2021). However, measures restricting the spread of COVID-19 have led to a decline in physical activity for most children and adolescents, especially among boys and older children and adolescents (Yomoda and Kurita, 2021). Compared with the first wave of the COVID-19 pandemic, physical activities in the second wave dropped by 3.1 percentage points (Gupta et al., 2021). Quarantine or self-isolation further reduces the amount and intensity of physical activities (Ammar et al., 2021), resulting in a general increase in inactivity and sedentary behavior (Schuch et al., 2020). On the other hand, the pandemic has triggered people to engage more in various activities to enhance mental health, but the number of people participating in physical exercise decreased, and the number of people participating in other activities increased (Gupta et al., 2021). Especially in the period of stricter prevention and control, the number of people reporting participation in camping, other family activities (such as sewing, puzzles and board games), and virtual group activities such as virtual churches has increased (Gupta et al., 2021). Therefore, it is imperative to address the following questions: Do physical exercises at home still maintain and/or improve the level of mental health? What range and types of sports activities do people usually engage in during a pandemic? What is the relationship between the intensity and frequency of indoor physical exercise and the improvement of mental health?

This article aims to address the above questions by building an integrated conceptual model and proposing directions for future research. It contributes to the literature by synthesizing the latest empirical findings since the outbreak of the COVID-19 pandemic in an integrated framework and providing a conceptual analysis of physical activity and mental health in times of a global pandemic.

## The Mechanisms of Physical Activity and Mental Health

Scholars have suggested various mechanisms that could potentially explain the positive effect of physical activity on mental health (Anderson and Shivakumar, 2013; Stubbs et al., 2017). Studies have revealed a variety of potential physiological mechanisms. For instance, it is well-acknowledged that, in addition to the improvement of physical health (Warburton et al., 2006), exercise may reduce stress hormones in the body, such as cortisol and adrenaline, and increases the levels of endorphins, which make people feel happy, optimistic and relaxed (Harber and Sutton, 1984). Moreover, exercise increases BDNF, which may help relieve the anxiety symptoms (Asmundson et al., 2013).

From the psychological perspective, there are three potential mechanisms. First, regular exercise increases the exposure to anxiety-related sensations, which helps to reduce anxiety sensitivity, resulting in a better mental health condition (Smits et al., 2008). Second, exercise may help build a sense of self-efficacy, improve self-image and confidence, which enhance the sense of control over stressful situations (Petruzzello et al., 1991). Third, exercise provides a distraction from stressors to enjoy the moment of physical activity, thus effectively reduce anxiety (Petruzzello et al., 1991).

## Coping Behaviors

Coping behaviors in this article refer to what people do in response to the stressful situation during COVID-19 (Cypryańska and Nezlek, 2020), and engaging in various types of physical activities is considered as a coping strategy to mitigate the negative effect on mental health and enhance well-being (Faulkner et al., 2020). According to the stress coping framework proposed by Lazarus and Folkman (1984), when people recognize the existence of a threat, they will be involved in either emotion-focused or problem-focused coping. Engaging in physical is a problem-focused, active coping, as people attempt to lessen the impact of pandemic-related stress. Studies have shown that fear and anxiety may trigger coping behaviors (Teasdale et al., 2012; Jin et al., 2020), which include following the public authority's preventive guidelines, such as wearing a facemask, washing hands, social distancing, and active engagement in physical exercises (Harper et al., 2020; Winter et al., 2020).

Lockdown during the pandemic has greatly reduced the outdoor activity time, and the outdoor activity space has been compressed, and people take home-based exercise as a coping strategy to promote psychological well-being (Faulkner et al., 2020; Vancini et al., 2021). The beneficial effects of home-based physical activities on mental health may be higher than other activities, such as religious activities, puzzles, and board games (Puyat et al., 2020; Gupta et al., 2021). Studies have focused on home-based physical exercise models that can be carried out by the general population to promote mental health from different perspectives.

Home-based exercise provides a safe alternative to public gyms and individual group fitness classes (Gupta et al., 2021). Studies have reported on the positive effects of family-based exercise programs on the anxiety, mood, social and emotional health of elderly cancer patients (Loh et al., 2019). Home exercise can include aerobic activities such as dancing (Gupta et al., 2021), balance and flexibility training, such as yoga (Ransing et al., 2020), muscle strength exercise, such as weightlifting (Gupta et al., 2021) endurance training and others (de Almeida et al., 2020). For example, as one of the common ways of indoor physical exercise, yoga exercise during the epidemic can reduce the perceived stress (Büssing et al., 2012), enhance emotional control and improve self-efficacy (Wang and Szabo, 2020), self-confidence and overall quality of life. Yoga is considered a non-pharmacological active intervention for mental problems (such as stress, fear) and mental disorders. It can be used alone or in combination with other interventions (Ransing et al., 2020), and it is one of the important measures to prevent or control mental health problems during the epidemic.

Exergames that use electronic products for human-computer interaction (such as simulated swimming, boating, cycling, running, and walking) may also help maintain and/or improve physical and mental health during the pandemic (Viana and de Lira, 2020). These active video games use visual and auditory stimuli to allow individuals to perform physical activity through interaction with motion and motion sensors (Viana and de Lira, 2020). Studies have shown that exergames have a positive effect on improving mood disorders (Byrne and Kim, 2019). Healthy young women performing a 20-min exergame Zumba fitness training at moderate intensity can reduce the level of anxiety (Viana et al., 2017).

## An Integrated Conceptual Framework

The control measures to limit the spread of the pandemic, such as lockdown, quarantine, self-isolation, social distancing, and wearing of masks have restricted both indoor and outdoor sports and fitness activities (Giustino et al., 2020; Schuch et al., 2020). Under the influence of various factors such as the general lack of time and space and the increase in family roles (Lum and Simpson, 2021), the types of physical activities that people can participate in have changed (Gupta et al., 2021). People's engagement in exercise has also changed (Faulkner et al., 2021). Generally, there are three key observations on the impact of the COVID-19 pandemic on physical exercise: (a) the pandemic triggers people's participation in physical activity; (b) the pandemic control measures inhibit physical activity, and (c) physical exercise helps improve the mental health and immune system (Constandt et al., 2020; Cheval et al., 2021; Maltagliati et al., 2021).

First, while the pandemic has brought a global disaster, it has triggered a new movement of people engaging in high-quality physical exercise. The information of physical activity benefits has effectively reached more people, which has become an opportunity for the education and development of the physical exercise system (Li and Liu, 2020). Government departments, sports companies, schools, families, opinion leaders, and ordinary citizens have been involved in the discussion of the benefits of sports and other physical activities (Faulkner et al., 2020; Li and Liu, 2020). The lack of exercise at home and the common awareness of improving immunity during the pandemic have prompted a large number of people to pay more attention to sports and exercise (Zhong et al., 2020). The pandemic has also greatly changed people's traditional views that sports and exercise must rely on professional venues, facilities and mentoring and that these activities can be carried out at home and with family members (Zhong et al., 2020).

Second, there are many difficulties and challenges for people to engage in physical exercise because of the Covid-related restrictions. The pandemic and its infection control measures have restricted people's participation in collective sports. Reduced physical activity during the pandemic has triggered more mental health problems (Gupta et al., 2021). Moreover, the economic and financial distress resulted from the pandemic may distract people from engaging in physical exercise (Salameh et al., 2020). Physical education faces new major challenges: students' home exercise may not reach the level required, online sports games lack physical and face-to-face social interaction Yuan (Li and Yuan, 2020). In colleges and universities, this situation is not optimistic. The attitude toward exercise is not positive enough; there is a significant gap between girls and boys in terms of physical exercise; the level of physical self-esteem of girls is relatively low (Li and Yuan, 2020).

Third, despite the challenges, studies on physical exercise during the pandemic confirm the positive impact of physical exercise on mental health and the immune system. Regular physical exercise during the pandemic can relieve psychological stress, improve the public's mental state, and effectively enhance the immune systems (Lou and Yan, 2020; Vancini et al., 2021). Scholars have called for active physical exercise to improve physical fitness, relieve stress, and promote physical and mental health (Constandt et al., 2020; Cheval et al., 2021; Maltagliati et al., 2021).

**Figure 1:**
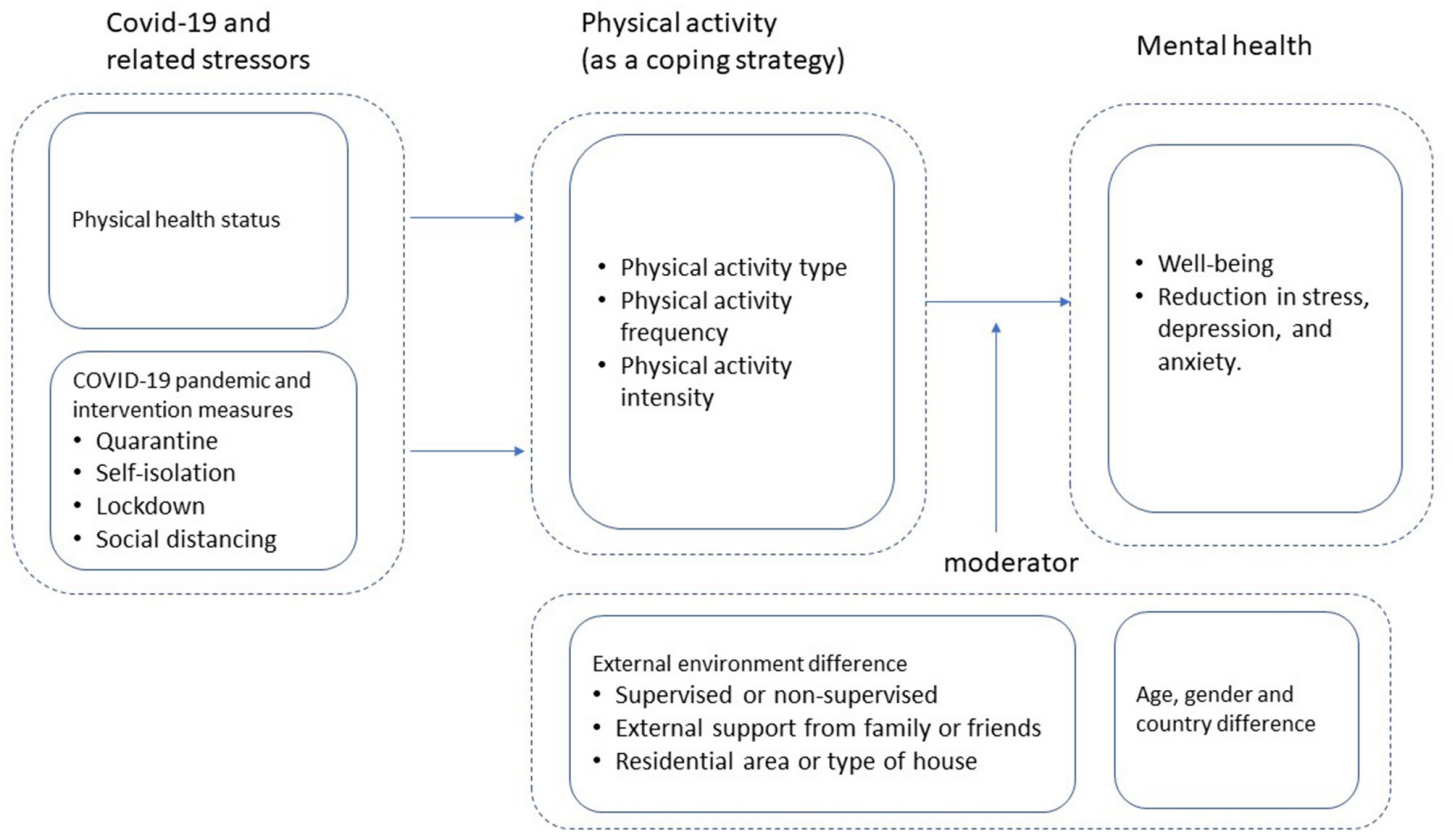
Integrated conceptual framework.

We synthesize the major thinking and findings in the extant literature in a conceptual model as presented in Figure 1, which depicts the relationships between measures to control the spread of COVID-19, physical activities and the deterioration of mental health, and the major mediating and moderating factors.

### Antecedents: Factors Hindering Physical Activity

Physical activity during the pandemic can reduce psychological pressure, promote physical and mental health, and improve the quality of life (Lum and Simpson, 2021). Both planned and unplanned physical activities are important explanatory variables for mental health, whether it is before or during the lockdown (Bird et al., 2021). We first explore the factors that hinder routine sports or activities during the COVID-19 lockdown.

#### Existing Health Status

Lack of physical exercise during the pandemic is likely to cause physical health problems. Sedentary behavior and physical inactivity often cause chronic and high-level inflammation (Zbinden-Foncea et al., 2020), which may make people more susceptible to the most severe form of COVID-19 (Vancini et al., 2021). Studies have shown that people with immunosuppression and chronic low-grade inflammation (especially the elderly) tend to have more severe forms of disease and higher mortality when infected with the COVID-19 virus (Yang et al., 2020). Therefore, moderate physical exercise is particularly helpful, as it can improve immunity and provide immune protection (Vancini et al., 2021). Unfortunately, existing poor health conditions may hinder physical activity (Lum and Simpson, 2021), thereby further increase the risk of infection. The level of severity of the illness to be high if infected, causing even more serious health problems.

#### Quarantine, Self-Isolation, and Lockdown

The lack of facilities and time constraints caused by quarantine, self-isolation and lockdown are important factors hindering the development of sports and other activities (Lum and Simpson, 2021). Along with the lengthening of the pandemic lockdown cycle, non-physical exercises such as positive thinking exercises, meditation and art, and light physical activities such as walking became more likely to be engaged in, while heavy physical activities such as cycling, hiking and jogging were substantially less likely to be engaged in Gupta et al. (2021). Motivation to exercise for some people may be weakened because of quarantine, self-isolation or lockdown (Maugeri et al., 2020). It is reported that the total physical activity of the population is significantly reduced during periods of quarantine, self-isolation and lockdown, with profound negative effects on people's mental health and well-being (Maugeri et al., 2020).

#### Social Distancing

Maintaining social distance is an important intervention to combat the spread of the virus because of the high infectivity and pathogenicity of COVID-19 (Brooks et al., 2020). However, the social distancing measure itself is not conducive to mental health (Salari et al., 2020), because of the reduced level of face-to-face physical activity and group physical activity (Moreira-Neto et al., 2021). The inhibited social interaction brought by social distancing may exacerbate the psychological distress brought by the pandemic (Carvalho Aguiar Melo and de Sousa Soares, 2020).

### Physical Activity Type, Intensity, and Frequency

The restricted collective activities during the pandemic have triggered mental health problems (Gupta et al., 2021), which heighten the importance of physical activity through alternative forms of exercise (Antunes and Frontini, 2021), especially, home-based exercise has been very popular as a coping strategy in such difficult times.

#### Types of Physical Activity

Nearly all types of physical activity are helpful and both physical exercise training and relaxation training can buffer the negative effects of stress on general health and mental health (Klaperski and Fuchs, 2021). Participation in physical activities at home such as yoga, dancing to music, exergames and other home-based exercises have been shown to alleviate a wide range of mental illness symptoms, improve anxiety, mood, social and emotional health (Chtourou et al., 2020; Puyat et al., 2020). Yoga, an ancient exercise focusing on both body and mind, is found to be very effective for improving mental well-being, through both psychological reappraisal and autonomic stress coping behaviors (Kinser et al., 2012). Wang and Szabo (2020) suggest that nearly all types of yoga are beneficial for the relief of stress. Exergames, which are exercises based on video games, appeal to the younger population to exercise at home, while the internet may further enable social interaction with friends remotely (Viana and de Lira, 2020).

#### Frequency of Physical Activity

The frequency of physical activity may not have the same degree of positive impact on mental health during the COVID-19 pandemic, while more physical activity is likely to be associated with higher levels of mental health (Maugeri et al., 2020). The number of participants participating in exercise is a moderator of the relationship between stress and overall health, and physical health alone cannot moderate the relationship between stress and mental health (Klaperski and Fuchs, 2021). People who become inactive have higher levels of loneliness, sadness and anxiety compared to those who are consistently active in physical activity (Werneck et al., 2021). Those who participated in at least one physical activity had significantly lower depression, anxiety, and psychological distress scores, compared to those who did not participate in physical activity (Gupta et al., 2021). People who did not exercise for a long time had a higher chance of experiencing loneliness and sadness (Werneck et al., 2021).

#### Physical Activity Intensity

The impact of physical activity intensity on mental health varies. Individuals who engaged in more active exercise behaviors during the pandemic reported better mental health and well-being (Faulkner et al., 2021). However, the step counting program alone was not associated with mental health scores (Bird et al., 2021). For those participants who participated in moderate to vigorous exercise (≥30 min) or vigorous exercise (≥15 min) daily, the level of anxiety and depression and the concurrent occurrence of depressive and anxiety were almost lower (Schuch et al., 2020). Physical exercise should be adjusted according to the health level of the participants and that a progressive intensity and a training volume model should be adopted (Chtourou et al., 2020).

### Moderators

#### Gender, Age, and Country Differences

##### Gender

There is a gender difference in exercise behaviors during the COVID-19 pandemic. It was found that women's exercise behaviors became more active during the COVID-19 pandemic (Faulkner et al., 2021). In general, there is a general reduction in exercise behavior caused, the reduction was greater for the male than the female group (Giustino et al., 2020).

##### Age

The COVID-19 pandemic may have different effects on exercise behaviors by different age groups. It was found that the exercise behavior of young people between 18 and 29 years old became more passive (Faulkner et al., 2021). In order to overcome the impact of COVID-19 on mental health, exercise intensity should be tailored for different ages. Adults must accumulate at least 75 min of high-intensity activities and 150 min of moderate-intensity activities each week. Children and adolescents who actively play at home or around the family can reduce the amount of training by 30% (Chtourou et al., 2020).

##### Country

Faulkner et al. (2021) compared the differences in exercise behaviors in the United Kingdom, Ireland, New Zealand, and Australia and their impact on mental health and well-being, and found that physical activity in different countries did not very much, but the level of mental health seems higher in New Zealand than in the other countries in their survey (Faulkner et al., 2021).

#### Contextual Variables

The major contextual variables include exercise monitoring (or supervising and mentoring), support from family and friends, and the place of residence.

##### Monitoring

Ideally, a physical activity program should be evidence-based, self-sustaining, feasible, acceptable, appropriate, and not dependent on remote or face-to-face supervision (Ransing et al., 2020). However, compared with self-directed exercise, supervised health care is more likely to stimulate exercise, thereby reducing self-reported depressive symptoms (Moreira-Neto et al., 2021). However, the pandemic restrictions make face-to-face supervision infeasible, and remote supervision has become a viable alternative (Moreira-Neto et al., 2021). Even home-based exercise requires mentoring programs such as smartphone applications and wearable sensors for monitoring (Chtourou et al., 2020) to help optimize activities, to achieve the desired benefits and minimize the potential risks that occur when certain activities are excessive or incorrectly performed (Puyat et al., 2020).

##### Support From Family or Friends

Support from family and friends are important for the engagement of physical activity during the outbreak. Women, especially those going through menopause, can increase their physical activity levels by making plans, setting goals, and exercising with friends or family (Lum and Simpson, 2021). Moreover, parental support may help children maintain or increase their physical activity levels during a pandemic (Yomoda and Kurita, 2021).

##### The Place of Residence

The location of residence had an impact on the practice of physical activity during the epidemic. Studies have shown that the decline in physical activity during the pandemic was smaller among children living in detached houses, houses with more space, rural areas, and those with more family members (Yomoda and Kurita, 2021).

## Future Research Directions

Physical activity plays an important role in maintaining and/or improving mental health. Future studies will generate more insights through the collection of data from large sample sizes, the use of cross-cultural contexts with adequate control for confounding factors, randomized controlled trials, and the adoption of more robust and thorough statistical analyses. Cross-cultural, multicenter studies using the same intervention and clearly defined components (currently missing) should be encouraged to explore the feasibility of physical activity as an effective intervention to maintain and improve mental health.

### Physical Activities in Times of Pandemic

Further research is needed to understand how to maintain physical activity during a major public health crisis. For example, given the various pandemic restriction measures, what physical activities are more conducive to people's physical and mental health? Which physical activities do people prefer to engage in to promote physical and mental health? How can the pandemic be used as an opportunity to further increase the awareness of the importance of physical activity? Physical exercise is often seen as a form of stress management, but causal evidence on the stress-buffering potential of exercise remains very limited (Klaperski and Fuchs, 2021). Additional research is needed to further explore the stress-buffering potential of physical exercise.

### Mediating and Moderating Effects

Future studies could further explore the mediating and moderating effects between physical activity and mental health in the context of major public health events.

#### Demographic Variables

Future research could focus on different gender and age groups to explore the motivations that motivate these groups to participate in physical activity during the pandemic and the magnitude of the moderating effects of gender and age in the relationship between physical activity and mental health. Moreover, the effects of different income levels and different racial/ethnic or cultural differences could be explored. Research can target specific groups (such as men, young people) that are most vulnerable to the negative effects of restriction measures (Faulkner et al., 2021), and explore instrumental measures to encourage physical exercise.

#### Country Context

Future research could compare the impacts of public health restrictions on exercise behaviors, and subsequently, mental health in different countries, especially those with different national cultures and income levels. Online supervised exercise has been proven to help improve mental health during the pandemic, however, it is not possible for some low- and middle-income countries to widely adopt this mode of exercise due to the lack of proper resources, especially internet access (Ransing et al., 2020). Therefore, further research is required to explore alternative measures to increase the level of physical exercise in different country contexts.

#### Modes of Exercise

The type of physical activity may moderate the relationship between exercise and mental health outcomes (Gupta et al., 2021). The amount of time required and the levels of intensity vary across the types of exercise, future research should compare the stress-buffering effects of different exercise types and the effects of alternative stress-modifying interventions to determine which interventions are most effective for different groups of people under different contexts (Klaperski and Fuchs, 2021). Future research may explore how suitable home-based exercise programs such as yoga (Ransing et al., 2020), non-pharmacological interventions such as home-based aerobics should be used in conjunction to address mental health issues during a pandemic. The effectiveness of physical activity in comparison with other leisure activities could also be examined.

#### Exercise Monitoring/Mentoring Modes

Some studies have found that supervised exercise is more motivating than self-directed exercise, thereby reducing depressive symptoms (Moreira-Neto et al., 2021). However, little is known about the motivation for the different modes of exercise. Therefore, future studies may examine motivation as a moderating factor to explain people's engagement in certain modes of exercise and the subsequent effects on mental health.

### Measures for Promoting Physical Activities

The extant literature is rather silent on strategies or concrete measures to encourage various physical activities during times of health crisis such as COVID-19. Yarimkaya and Esentürk (2020) recommended several strategies that families could adopt to help children with autism spectrum disorders practice physical activity during the pandemic. However, strategies of physical activity for the general healthy population are also urgently needed during this difficult time. Further research is required to test the effectiveness of the various recommended strategies and their suitability for the general public.

## Conclusions

This article explores the impact of the COVID-19 pandemic on physical activities and the evidence that physical activities can maintain and/or improve mental health based on a review of the literature on physical exercise and mental health published between 2019 and 2021. The empirical evidence reported in those studies shows that in the context of COVID-19, maintaining regular exercise is effective, and engaging in alternative modes of physical activity is a key strategy to maintain mental health and well-being (Maugeri et al., 2020). Further research is called for to contribute to the development and implementation of public health interventions to encourage various physical activities across different social groups in different contexts.

## Author Contributions

XA, XW, and ZL contributed to the general conception and the conceptual framework of the study. XA, XW, JY, and ZL took part in collecting relevant data (including research findings) and worked together to complete the first draft of the manuscript. Analysis of the data on which conclusion was based were mainly done by XA, XW, and ZL. XA and ZL revised and finalized the manuscript. All authors contributed to the article and approved the submitted version.

## Funding

This research has received fund from Wuhan Sports University's East Lake Scholar Research Fund National Social Science Foundation of China (Grant no. 20ZDA067).

## Conflict of Interest

The authors declare that the research was conducted in the absence of any commercial or financial relationships that could be construed as a potential conflict of interest.

## Publisher's Note

All claims expressed in this article are solely those of the authors and do not necessarily represent those of their affiliated organizations, or those of the publisher, the editors and the reviewers. Any product that may be evaluated in this article, or claim that may be made by its manufacturer, is not guaranteed or endorsed by the publisher.
